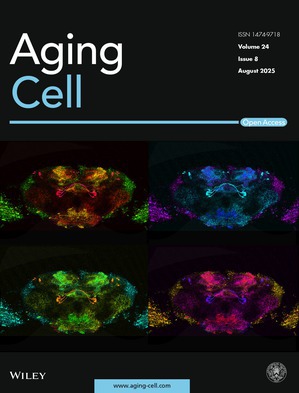# Additional Cover

**DOI:** 10.1111/acel.70199

**Published:** 2025-08-12

**Authors:** Silas A. Buck, Samuel J. Mabry, Tenzin Kunkhyen, Zilu Yang, Sophie A. Rubin, Jinting Yang, Claire E. J. Cheetham, Zachary Freyberg

## Abstract

Cover legend: The cover image is based on the article *dVGLUT Is a Mediator of Sex Differences in Dopamine Neuron Mitochondrial Function Across Aging and in a Parkinson's Disease Model* by Silas A. Buck et al., https://doi.org/10.1111/acel.70096.